# Numerical Study of Temperature Fields and Control Methods for Improving the Grain Storage Safety of Semi-Underground Granaries

**DOI:** 10.3390/ma19153357

**Published:** 2026-08-06

**Authors:** Haitao Wang, Jiabao Liu, Liu Yang, Kai Liu, Shujie Niu, Yuanyuan Wang

**Affiliations:** 1 College of Civil Engineering, Henan University of Technology, Zhengzhou 450001, China; liujiabao@stu.haut.edu.cn (J.L.); 18623717275@163.com (K.L.); wangyuanyuan@stu.haut.edu.cn (Y.W.); 2 Henan University of Technology Design and Research Co., Ltd., Zhengzhou 450001, China

**Keywords:** semi-underground granary, geothermal energy, temperature field, temperature control method, embedded-pipe wall

## Abstract

The semi-underground granary is a new type of energy-saving grain storage facility that can use shallow geothermal energy to reduce energy consumption during grain storage. However, unclear temperature fields and the lack of grain pile temperature control methods are not conducive to the design and application of semi-underground granaries. In this study, the temperature fields and temperature control methods for grain piles in a semi-underground granary were numerically investigated by using an experimentally verified COMSOL model and a collaborative simulation method combining steady-state heat transfer and dynamic heat transfer. Multiple grain storage temperature control methods for the semi-underground granary were presented to improve grain storage safety, including an intermediate floor slab, an embedded-pipe wall, floor burial depth, and envelope insulation. The results showed that there was significant spatial heterogeneity in the temperature field distribution of the grain pile in the semi-underground granary. The large thermal inertia of the soil and the stable low-temperature soil environment reduced the influence of outdoor air temperature variations on the grain pile temperature field. Installing an intermediate floor slab could achieve natural low-temperature grain storage in the underground section of the semi-underground granary. An embedded-pipe wall could effectively solve the problem of local temperature increases in grain piles caused by heat transfer through the granary walls. The floor burial depth of the semi-underground granary was a key influencing factor of heat transfer through the granary wall. Granary wall thickness had a significant impact on the thermal performance of the walls and the grain pile temperature field due to changes in wall insulation. These results can provide beneficial suggestions for guiding the design of grain storage temperature control methods in semi-underground granaries.

## 1. Introduction

Food security and energy conservation in the building sector are important national strategies in China, and developing advanced green, energy-efficient grain storage technologies is a key pathway to achieving these strategic goals. The semi-underground granary is a new type of grain storage facility that utilizes shallow geothermal energy to achieve green, ecological, and energy-efficient grain storage. Grain temperature is very important for grain storage safety [1]. The temperature distribution characteristics of grain piles in semi-underground granaries under dynamic outdoor thermal environmental conditions are highly complex. The external environment of a semi-underground granary is composed of soil and outdoor air. The interior of a semi-underground granary consists of the grain pile and the air layer above it. The temperature of grain piles in semi-underground granaries is influenced by the coupled effects of outdoor air temperature and soil temperature. The study of heat transfer in the envelope structure of a semi-underground granary is a fundamental basis for thermal optimization design and grain storage thermal environment control [2]. Therefore, it is very important to study the temperature field distributions and temperature control methods of grain piles in semi-underground granaries.

Extensive research has been conducted on the envelope heat transfer and temperature field distribution of grain piles in traditional above-ground granaries. For example, Wang et al. [3] investigated the relationship between the average temperature of the grain pile and the daily ambient air temperature through numerical simulation and experimental methods. Quemada-Villagómez et al. [4] investigated the influence of outdoor environmental temperature on grain pile temperature inside silos using numerical simulation, revealing that high outdoor temperatures can cause internal silo temperatures to reach as high as 42 °C. Rusinek et al. [5] established a discrete element method (DEM) numerical model to simulate the self-heating process of rapeseed in silos. The numerical simulation was used to investigate the temperature distribution and predict self-heating zones, revealing that the maximum detection distance from the heat source to the farthest sensor ranged from 0.5 m to 0.6 m. Chen et al. [6] developed a heat and mass transfer model for the hot-air drying process of wheat grain piles in granaries. They investigated the internal thermo-humid transfer characteristics under a specific operating condition, revealing a non-uniform temperature distribution within the grain pile under this condition. The wheat grain pile accumulated heat in the upper region because of the lower thermal diffusivity of the grain pile.

Underground granaries can utilize shallow geothermal energy to achieve green, ecological, and energy-efficient grain storage [7,8]. Underground granaries have advantages in energy efficiency, land conservation, and safety [9,10]. The study of envelope heat transfer theory for underground granaries is the foundation for thermal optimization design and grain storage temperature control methods in underground granaries. Chen et al. [11] used numerical simulation to investigate the effects of local heat sources on temperature variations within the grain pile in an underground granary. They found that a local high-temperature zone gradually formed in the middle-upper layer of the grain pile during the winter static storage stage. Zhang et al. [12] investigated the variation characteristics of the temperature field in grain piles during static storage in a plastic-lined underground granary using experimental and numerical simulation methods. They found that the heat effect of grain respiration was intense during static storage. The bottom of the grain pile warmed up first and then formed a high-temperature thermal core within the grain pile in the underground granary. The temperature in the upper area of the grain pile was susceptible to the outdoor thermal environment, while the temperature in the middle and bottom zones of the grain pile was relatively stable.

Low-temperature grain storage is helpful to maintain grain quality and reduce grain storage losses [13,14]. Grain storage temperature control methods are an intensive research area in energy-saving and low-carbon grain storage. Granary mechanical ventilation, embedded pipe envelopes [15], double-layer ventilated roofs [15,16], high-reflectivity coatings [17,18], and exterior surface radiative cooling [19,20] have been applied in grain storage facilities for energy saving and carbon emission reduction. Underground and semi-underground granaries can use shallow geothermal energy to achieve natural low-temperature grain storage. The external environment of a semi-underground granary is composed of soil and outdoor air. The temperature of grain piles in semi-underground granaries is influenced by the coupled effects of outdoor air temperature and soil temperature. There are strong coupled effects between outdoor air temperature and soil temperature. Therefore, these effects should be considered in calculating the heat transfer through the semi-underground granary envelope. The total heat transfer through the semi-underground granary envelope is not simply the sum of heat transfer of the above-ground envelope and the underground envelope.

Existing studies have focused on envelope heat transfer, grain pile temperature field, and indoor thermal environment control of above-ground and underground granaries [21]. However, some key issues in the thermal design of envelopes for semi-underground granaries remain unclear [22]. First, the external environment of a semi-underground granary is composed of soil and outdoor air. The external environment of a semi-underground granary is different from those of underground and above-underground granaries. Envelope heat transfer in a semi-underground granary is influenced by the coupled effects of outdoor air temperature, soil temperature, and indoor environmental temperature. Complex heat transfer boundary conditions and numerous influencing factors result in unclear thermal characteristics of the semi-underground granary envelope [23]. Second, existing studies of the grain pile temperature distributions in semi-underground granaries usually assumed that the soil temperature was constant [24]. The dynamic effects of outdoor environment fluctuations on grain pile temperature distributions in semi-underground granaries were neglected. This makes it difficult to accurately predict the actual thermal environmental changes in a semi-underground granary during grain storage. Finally, grain storage temperature control methods for above-ground granaries cannot be directly applied to semi-underground granaries [25,26]. Semi-underground granaries still lack mature and efficient temperature control methods for grain piles. The lack of temperature control methods for grain piles is detrimental to grain storage safety and energy-saving in semi-underground granaries.

To solve the above problems, this study will numerically investigate grain pile temperature distribution and grain storage temperature control methods for semi-underground granaries. The innovations of this study are to (1) investigate temperature fields of grain piles in semi-underground granaries by using a collaborative simulation method combining steady-state heat transfer and dynamic heat transfer; (2) propose an intermediate floor slab-based grain temperature control method for semi-underground granaries to achieve natural low-temperature grain storage; (3) present an efficient embedded-pipe wall-based local high-temperature control method for grain piles in semi-underground granaries to reduce peak grain storage temperatures and improve grain storage safety; (4) discuss the floor burial depth-based grain temperature control method and envelope insulation-based grain temperature control method for semi-underground granaries.

## 2. Materials and Methodology

### 2.1. Numerical Model of the Semi-Underground Granary

**Figure 1:**
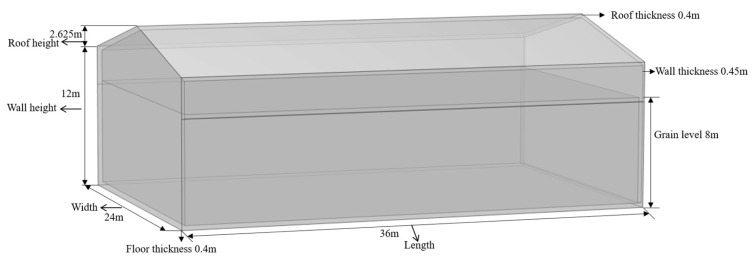
Numerical COMSOL model of the semi-underground granary.

**Figure 2:**
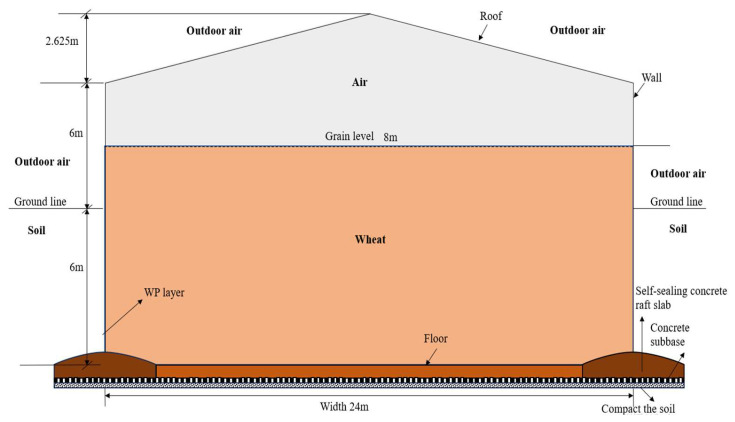
Structural diagram of the semi-underground granary.

A three-dimensional numerical model of a semi-underground granary was developed in the COMSOL multi-field coupled simulation software to investigate the temperature fields and storage grain temperature control methods for semi-underground granaries. The COMSOL software 5.6 was developed in Stockholm, Sweden. The three-dimensional COMSOL model of a semi-underground granary was built based on a real granary in Zhengzhou city in China. The granary concerned was a large warehouse, as shown in Figure 1. The granary had a storage capacity of 4970.16 tons. The granary was 36 m in length, 24 m in width, and 12 m in height. The granary roof was a sloped roof with a height of 2.625 m. The above-ground section of the granary wall was 6 m high, and the underground section was 6 m high. The height of the grain pile in the granary was 8 m. The zone above the grain pile was an air layer. The granary wall consisted of a 20 mm cement mortar layer, a 5 mm waterproof membrane layer, a 400 mm reinforced concrete layer, and a 25 mm waterproof mortar layer. The granary roof consisted of a 20 mm cement mortar layer, a 5 mm waterproof membrane layer, a 350 mm reinforced concrete layer, and a 25 mm cement mortar layer. The granary floor consisted of a 20 mm cement mortar layer, a 5 mm waterproof membrane layer, a 500 mm reinforced concrete layer, and a 25 mm cement mortar layer. Figure 2 shows a structural diagram of the semi-underground granary. Table 1 lists the thermophysical parameters of the main building materials in this study. The thermal conductivity of wheat grain is lower than those of concrete and soil, as shown in Table 1. The thermal conductivity value for wheat refers to the effective thermal conductivity of the grain pile ($k_{\mathrm{eff}}$) [27]. The thermal conductivity of wheat grain is low, and its ventilation resistance is high. These properties of grain piles increase the difficulties associated with their temperature control.

### 2.2. Governing Equations of Heat Transfer in Semi-Underground Granary

#### 2.2.1. Solid Heat Transfer Control Equation

The heat transfer in roofs, walls and floors of a semi-underground granary is solid heat transfer. Because the roofs, walls and floors of the semi-underground granary have excellent waterproof performance, the water migration problem is not considered in the heat transfer processes of the semi-underground granary envelope. The main heat transfer mode of solid heat transfer is conductive heat transfer. The solid heat transfer control equation can be expressed as follows [30]:

(1) $$\rho_{\mathrm{so}}c_{p,\mathrm{so}}\frac{\partial T_{\mathrm{so}}}{\partial t}=\nabla \cdot \left(k_{\mathrm{so}}\nabla T_{\mathrm{so}}\right)$$

where $\rho_{\mathrm{so}}$ represents the density of solid materials, kg/m^3^; $c_{p,\mathrm{so}}$ represents the constant pressure specific heat capacity of the solid material, J/(kg⋅K); $k_{\mathrm{so}}$ represents the thermal conductivity of building solid materials, W/(m⋅K); $T_{\mathrm{so}}$ represents the temperature of the granary envelope, K; $\nabla T_{\mathrm{so}}$ represents the temperature gradient of the solid material of the envelope of the semi-underground granary.

#### 2.2.2. Heat Transfer Control Equation for Porous Media

Heat transfer in soils and grain piles is porous medium heat transfer. The equivalent physical parameters of the porous medium are used to calculate the heat transfer in the porous medium. Local heat balance energy conservation equations for a porous medium are used to simulate the heat transfer in the porous medium. Thermophysical parameters of soil and grain pile material are shown in Table 1. When convective heat transfer is ignored, the local heat balance energy conservation equations can be expressed as follows [31]:

(2) $$\rho_{\mathrm{eff}}c_{p,\mathrm{eff}}\frac{\partial T}{\partial t}=\nabla \cdot (k_{\mathrm{eff}}\nabla T)+Q_{\mathrm{src}}$$

(3) $$c_{p,\mathrm{eff}}=\frac{(1-\varepsilon )\rho_{s}c_{p,s}+\varepsilon \rho_{f}c_{p,f}}{\rho_{\mathrm{eff}}}$$

where $\rho_{\mathrm{eff}}$ represents the equivalent density of porous media, kg/m^3^; ε represents the porosity, $\rho_{s}$ represents the solid density, and $\rho_{f}$ represents the fluid density; $c_{p,\mathrm{eff}}$ represents the equivalent constant pressure specific heat capacity of porous media, J/(kg·K); $c_{p,s}$ represents the specific heat capacity of solids at constant pressure, J/(kg·K), $c_{p,f}$ represents the specific heat capacity of fluids at constant pressure; T represents the temperature of the porous medium, K; t represents time, and s represents the time variable of the heat transfer process; $\frac{\partial T}{\partial t}$ represents the rate of change in the temperature of porous media with time, K/s, which characterizes the transient change in the temperature field; $k_{\mathrm{eff}}$ represents the equivalent thermal conductivity of porous media, W/(m·K); ∇T represents the temperature gradient of porous media, K/m, which is the temperature potential difference driving the heat conduction process and determines the direction and size of heat flux density; $Q_{\mathrm{src}}$ denotes the volume heat source intensity in porous media, W/m^3^.

The effective thermal conductivity of the grain pile ($k_{\mathrm{eff}}$) is defined by using the standard volumetric averaging method as follows [32]:

(4) $$k_{\mathrm{eff}}=(1-\varepsilon )k_{s}+\varepsilon k_{f}$$

where $k_{s}$ represents the thermal conductivity of the grain material; $k_{f}$ represents the thermal conductivity of the fluid (air) within the pores. The values for $k_{s}$, $k_{f}$ and the porosity ε are determined according to Table 1 for the corresponding materials. The effective thermal conductivity of soil material can also be determined by using Equation (4).

#### 2.2.3. Fluid Heat Transfer Control Equation

The heat transfer of outdoor air and the air layer above a grain pile is fluid heat transfer. The fluid heat transfer control equation is a mathematical expression based on the three basic physical laws of mass conservation, momentum conservation and energy conservation. The following is the mass conservation equation, which can describe the mass conservation relationship of air fluid [33]:

(5) $$\frac{\partial \rho_{\mathrm{air}}}{\partial t}+\nabla \cdot \left(\rho_{\mathrm{air}}u\right)=0$$

where $\rho_{\mathrm{air}}$ denotes the density of air, kg/m^3^; t is the time, s; u denotes the velocity vector of air, m/s. In this study, air is considered as an incompressible fluid. Under the Boussinesq approximation, density variations are considered only in the buoyancy term.

The momentum conservation equation describes the evolution of the airflow velocity field, where the buoyancy term provides the driving force for natural convection. The following is the momentum conservation equation [33].

(6) $$\rho_{\mathrm{air}}\left(\frac{\partial u}{\partial t}+u\cdot \nabla u\right)=-\nabla p_{air}+\mu \nabla^{2}u+\rho_{\mathrm{air}}g$$

where $p_{air}$ represents the pressure, Pa; μ represents the dynamic viscosity of air, Pa s; $\nabla^{2}$ represents the Laplace operator; g represents the gravity acceleration vector, m/s^2^; $\rho_{\mathrm{air}}g$ represents volume force; $\rho_{\mathrm{air}}$ represents a function of temperature; $\rho_{0}$ represents the air density at the reference temperature $T_{0}$.

The energy conservation equation describes the evolution of the air temperature field, including the two effects of heat conduction and heat convection [33].

(7) $$\rho_{\mathrm{air}}c_{p,\mathrm{air}}\left(\frac{\partial T_{\mathrm{air}}}{\partial t}+u\cdot \nabla T_{\mathrm{air}}\right)=\nabla \cdot \left(k_{\mathrm{air}}\nabla T_{\mathrm{air}}\right)$$

where $c_{p,\mathrm{air}}$ represents the specific heat capacity of air at constant pressure, J/(kg·K); $k_{\mathrm{air}}$ represents the thermal conductivity of air, W/(m·K). $u\cdot \nabla T_{\mathrm{air}}$ represents the convection term reflecting the contribution of fluid motion to heat transport.

#### 2.2.4. Conjugate Heat Transfer Control Equation

Heat transfer between a solid surface and air in a semi-underground granary is conjugate heat transfer. Conjugate heat transfer is a combination of solid heat transfer and fluid heat transfer. The dominant heat transfer mode in the fluid side is convective heat transfer. The heat transfer in the air side can be expressed as follows [34]:

(8) $$\rho_{air}c_{p,\mathrm{air}}(\frac{\partial T_{air}}{\partial t}+(u\cdot \nabla )T_{air})=-(\nabla \cdot q_{air})+\tau S-{\left.\frac{T_{air}}{\rho_{air}}\frac{\partial p_{air}}{\partial T_{air}}\right|}_{P}(\frac{\partial p_{air}}{\partial t}+(u\cdot \nabla )p_{air})+Q_{air}$$

where $q_{air}$ represents the heat flux density of air, W/m^2^; $p_{air}$ represents the air pressure, Pa.

The heat transfer in the solid side can be expressed as follows [34]:

(9) $$\rho_{\mathrm{so}}c_{p,\mathrm{so}}\frac{\partial T_{so}}{\partial t}=-(\nabla \cdot q_{so})+Q_{so}+Q_{ted}$$

where $Q_{ted}$ represents the source of thermoelastic dissipation, W/m^3^; $q_{so}$ represents the heat flux density vector, W/m^2^.

The k−ε model in COMSOL software 5.6 is used to predict the flow field near the outside surface of a solid. The following equations can be used to calculate the heat transfer between the outside surface of the solid and air [34].

(10) $$q_{air-so}=\frac{\rho_{air}c_{p,\mathrm{air}}u^{*}(T_{so}-T_{air})}{T^{+}}$$

where $T_{air}$ represents the air temperature, K; $u^{*}$ represents the friction velocity; $T^{+}$ represents the dimensionless temperature.

In the process of conjugate heat transfer, the solid is regarded as an opaque medium. The air is regarded as a transparent medium. The radiation heat transfer between different solid surfaces through the air layer in the granary can be determined by the following equations [34].

(11) $$q_{ra}=\varepsilon n^{2}\sigma (T_{sou}^{4}-T_{tar}^{4})$$

where $q_{ra}$ represents the radiation heat transfer between two solid surfaces, W/m^2^; ε represents the thermal emissivity of a solid surface; n represents the refractive index of the air layer; σ represents the Stefan–Boltzmann constant; $T_{sou}$ denotes the outer surface temperature of the high-temperature solid during radiation heat transfer, K; $T_{tar}$ denotes the outer surface temperature of the low-temperature solid during radiation heat transfer, K.

The heat transfer from the sun to the ground surface can be calculated using the following equation [34]:

(12) $$Q_{so}=\alpha I_{total}A$$

where α represents the ground radiation heat transfer absorption coefficient; $I_{total}$ represents the intensity of solar radiation, W/m^2^; A represents the ground area, m^2^.

### 2.3. Initial and Boundary Conditions

Initial temperatures of the grain pile and air layer were set to 25 °C. The constant-temperature layer was located at a depth of 17 m. The temperature of the constant-temperature layer was 17 °C in Zhengzhou city in China [35]. The far-field boundary of the soil was set as an adiabatic boundary condition. To couple the fluid and solid domains, continuity of temperature and heat flux should be imposed at the interface between the grain pile and the granary envelope and the interface between the soil and the granary envelope. To couple the fluid and porous domains, continuity of temperature and heat flux should be imposed at the interface between the air and the grain pile. To couple the solid and porous domains, continuity of temperature and heat flux should be imposed at the interface between the granary envelope and the grain pile. The convective heat transfer coefficient of the granary wall outer surface was set to 23 W/(m^2^⋅K) [36]. Outdoor wind speed was set to 0 m/s. Outdoor air temperature was set to the typical annual outdoor air temperature of Zhengzhou city in China. The solar radiation heat absorption coefficient of the outer surface of the exterior wall of the granary was set to 0.6. The dynamic viscosity of air (μ) was set to 1.789 × 10^−5^ Pa⋅s. The Stefan–Boltzmann constant (σ) was set to 5.67 × 10^−8^ W/(m^2^⋅K^4^).

### 2.4. Multi-Field Coupled Simulation Approach

In this study, temperature fields and grain storage temperature control methods for semi-underground granaries were systematically studied using a new collaborative simulation method combining steady-state heat transfer and dynamic heat transfer. Using this method, steady-state heat transfer simulations were used to study floor burial depth-based and envelope insulation-based grain temperature control methods for semi-underground granaries. Steady-state heat transfer simulations can eliminate adverse effects of outdoor air temperature fluctuations on the simulation results. Transient heat transfer simulations were used to study the temperature fields of grain piles and floor slab-based and embedded-pipe wall-based grain temperature control methods for semi-underground granaries. The dynamic heat transfer simulations use outdoor thermal environment data as dynamic boundary conditions, which will bring the simulation results closer to the actual heat transfer behavior in a semi-underground granary. In this study, fluid–solid–heat-coupled heat transfer in the semi-underground granary was implemented by selecting appropriate physics interfaces and simulation equations in COMSOL software. The laminar flow interface was applied to the air layer. The porous media heat transfer interface was applied to the soil and grain pile. The solid heat transfer interface was applied to the concrete roofs, walls, and floors. These fluid, solid, and porous medium domains were coupled via the non-isothermal flow multiphysics node to achieve bidirectional temperature–velocity coupling. At the laminar flow interface, the air layer was treated as an incompressible flow with the Boussinesq approximation. This numerical model of the semi-underground granary included solid, fluid, and porous medium domains. The soil and grain bulk were considered as porous media. The heat transfer equations were solved separately for each domain, and thermal coupled at the domain interfaces was achieved through conjugate heat transfer conditions. The physics interfaces automatically identified different material regions in the computational domain and solve the energy equation in a unified manner. Through non-isothermal flow coupling, the temperature distribution obtained from the heat transfer interface was mapped to the Boussinesq buoyancy term in the laminar flow interface, which in turn drove the airflow. The non-isothermal flow node automatically applied temperature and heat flux continuity conditions at solid–fluid interfaces, eliminating the need for additional coupled boundaries. A segregated solver was adopted to decouple the velocity and temperature fields for efficient multiphysics solution, which in turn enhanced numerical stability for non-isothermal flows with the Boussinesq approximation. The Newton–Raphson iteration was applied to nonlinear convergence. In transient simulation study, the implicit backward differentiation formula (BDF) with adaptive time-stepping was used to accurately capture the smooth temporal evolution.

### 2.5. Cell Size Independence Test

**Figure 3:**
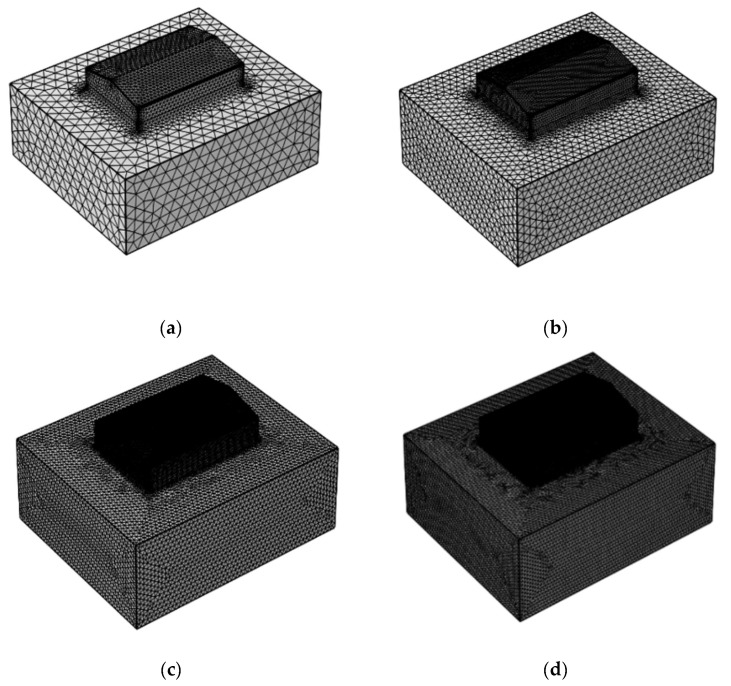
Different meshing schemes. (**a**) Mesh 1 (1,055,490 cells); (**b**) Mesh 2 (2,062,671 cells); (**c**) Mesh 3 (2,479,938 cells); (**d**) Mesh 4 (3,077,995 cells).

Grid independence verification was performed in this study to eliminate the influence of grid discretization on the calculation results. Further refinement of the grid produced no significant change in the key physical quantities, confirming that the results are grid-independent. As shown in Figure 3, four grids were generated with cell counts of 1,055,490 (Grid 1), 2,062,671 (Grid2), 2,479,938 (Grid 3), and 3,077,995 (Grid 4). Because the floor serves as a major heat-transfer pathway, the average temperature of the inner surface of the granary floor was selected as the monitored variable. All four grids were simulated under the same physical model, boundary conditions, and solver settings.

**Figure 4:**
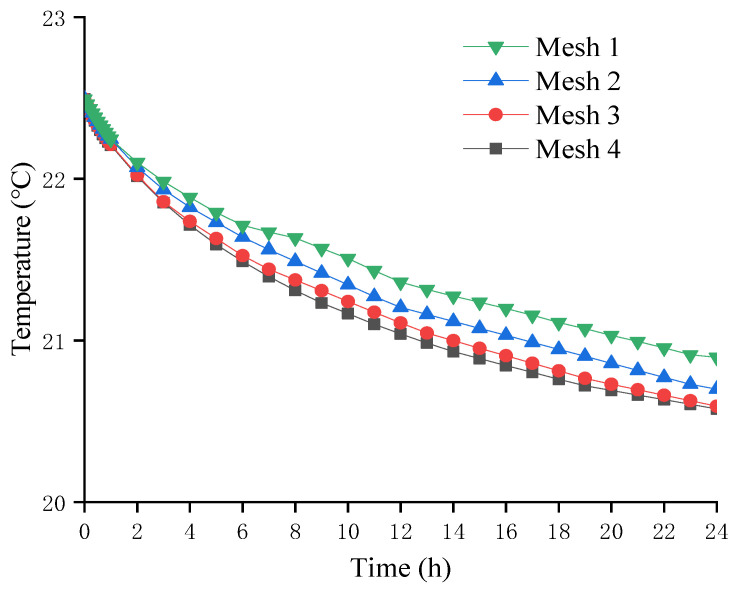
Mesh-size-independent test.

Figure 4 shows the simulated average inner surface temperature of the floor for four different grid schemes over a three-day transient heat transfer period. The temperature drops from 22.5 °C to about 21 °C on the second day. The phenomenon is attributed to the thermal equilibration between the uniform initial temperature field and the lower boundary temperatures. To evaluate the influence of grid refinement, the relative percentage change method was used to analyze the simulation results of different grid schemes. The simulation results of Grid 4 were used as the reference, as shown in Table 2. The relative error (RE) was calculated by using Equation (13).

(13) $$RE=\frac{\left|T_{\mathrm{mesh}}-T_{\mathrm{ref}}\right|}{T_{\mathrm{ref}}}\times 100\%$$

where RE represents relative percentage change, %; $T_{\mathrm{mesh}}$ represents temperature of coarser grids, K; $T_{\mathrm{ref}}$ represents temperature of reference grid, K.

### 2.6. Numerical Model Validation for Semi-Underground Granary

**Figure 5:**
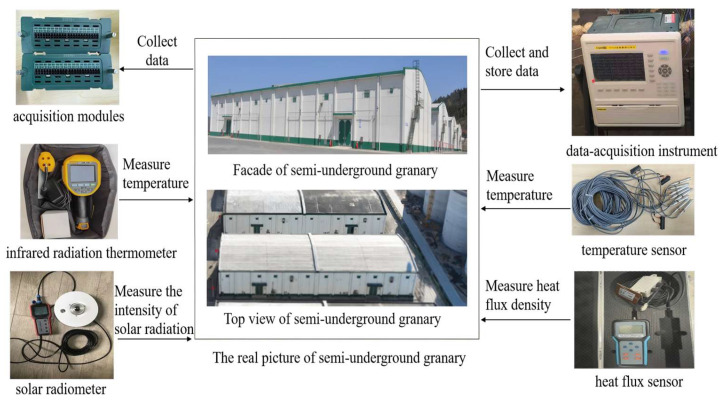
Underground granary and measuring equipment.

In this study, the prediction accuracy of the numerical heat transfer model for the semi-underground granary envelope was verified by using test data from an actual granary in Zhengzhou city in China (Figure 5). Grain pile temperature measured by Jin et al. [37,38] and measured hourly outdoor air temperature were used to validate the prediction accuracy of the numerical semi-underground granary model.

**Figure 6:**
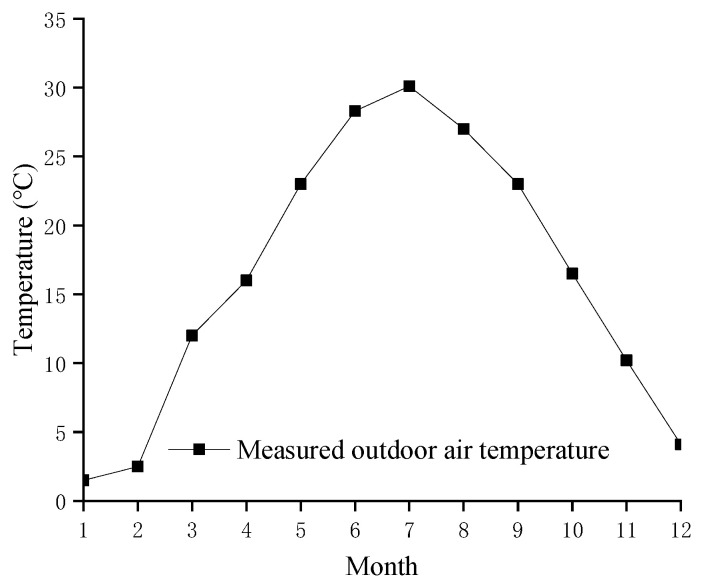
Measured outdoor air temperature.

**Figure 7:**
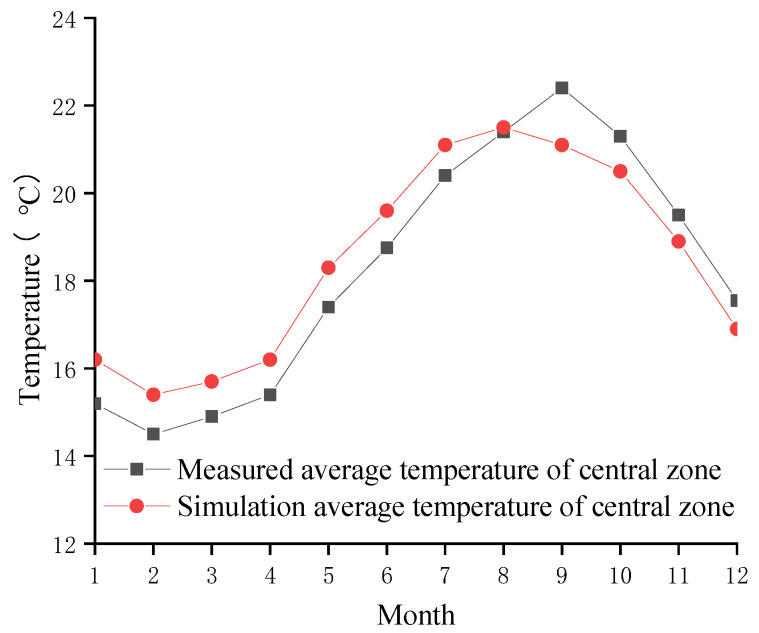
Measured and simulated average temperature of central zone in grain pile.

The numerical model of a semi-underground granary was validated by comparing the measured average temperature of the central zone in the grain pile with the simulated ones. Figure 6 shows the measured outdoor air temperature over an entire year. Figure 7 shows the measured and simulated average temperatures in the central zone in the grain pile. In the central zone of the grain pile in the experimental granary, temperature sensors were spaced at a 1 m interval along the height and a 1.5 m interval along the horizontal of the grain pile. Temperature sensors were installed on multiple vertical cables. The measured temperature of the central zone in the grain pile could be determined by using the average values of all corresponding temperature sensor readings. The simulated average temperature of the central zone in the grain pile agreed well with the measured average temperature. The lowest average temperature of the central zone in the grain pile occurred in February, and the highest average temperature occurred in September. The error between the simulated and measured average temperatures of the central zone in the grain pile was within 1.5 °C. The percentage error was less than 6%, which was within an acceptable range for engineering applications [39,40].

## 3. Temperature Fields of Grain Pile in Semi-Underground Granary

### 3.1. Temperature Field Distributions of Grain Pile in Semi-Underground Granary

**Figure 8:**
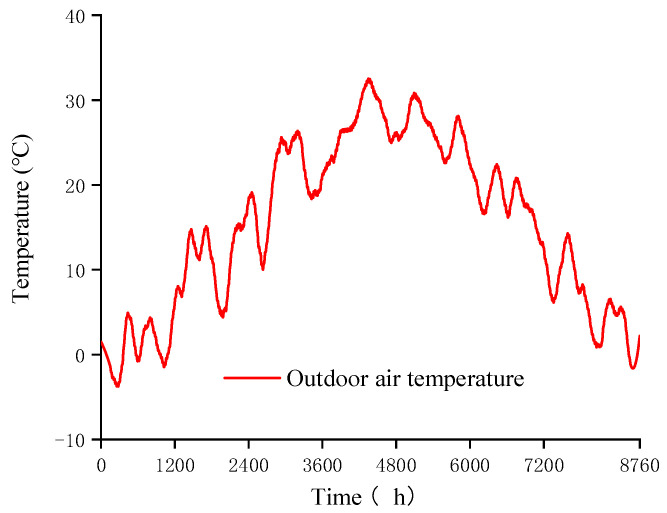
Typical annual outdoor air temperature for Zhengzhou city.

**Figure 9:**
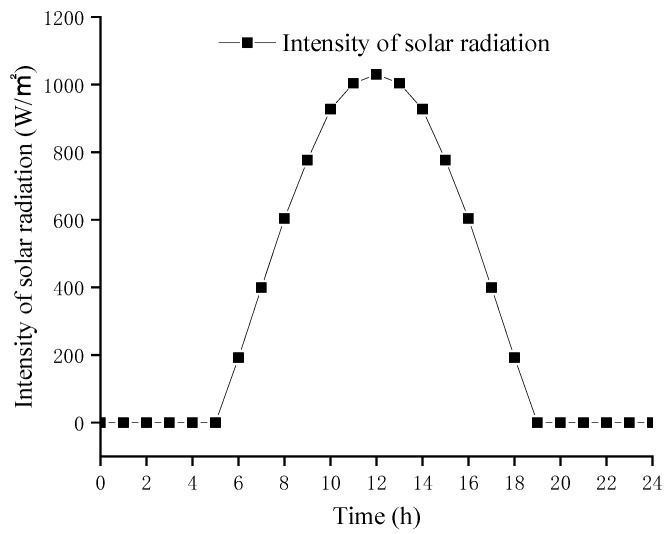
Typical hourly solar radiation intensity for Zhengzhou city.

The typical annual outdoor air temperature for Zhengzhou city in China and the semi-underground granary numerical model described in Section 2.1 were used to investigate the temperature field distributions of the grain pile in a semi-underground granary. Figure 8 shows the typical annual outdoor air temperature for Zhengzhou city in China. The initial temperatures of the grain pile and air layer above the grain plie were set to 25 °C. Figure 9 shows typical hourly solar radiation intensity for Zhengzhou city in China. The outdoor air wind speed was set to 0 m/s.

**Figure 10:**
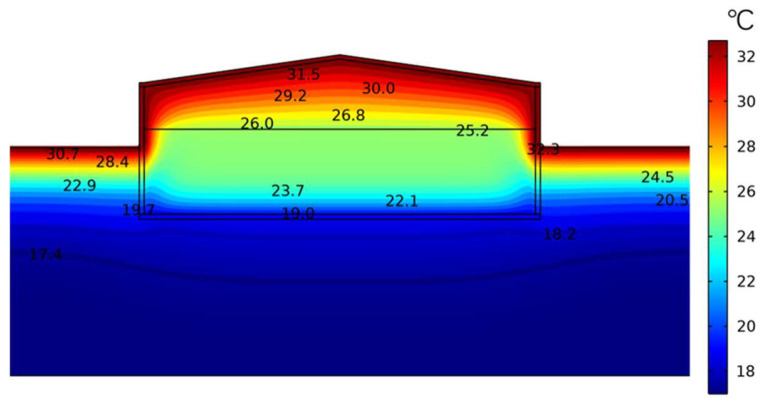
Simulated central vertical view of granary temperature field distributions for 17 August.

Figure 10 shows a central vertical view of the granary temperature field distributions for 17 August. The semi-underground granary exhibited a gradual temperature increase and temperature stratification phenomenon from the bottom to the top. The above-ground part of grain pile showed the temperature distribution phenomenon of hot skin and cold core in the grain pile due to granary envelope heat transfer. The highest temperature in the local zone of the grain pile near the ground surface was 32.3 °C. The temperatures of the grain pile in the zone near the ground surface had large temperature gradients, and large local temperature differences in the grain pile could increase the risk of condensation in the local zone near the granary wall. The temperature in the underground part of the grain pile remained low throughout the year. The highest temperature in the underground part of grain pile was 23.7 °C, which exceeded the low-temperature grain storage threshold of 20 °C [34,35,36]. Grain storage temperature is very important for grain storage safety. The lower the grain storage temperature, the safer the grain storage. Semi-underground granaries can use not only the stable low-temperature environment of the underground soils for low-temperature grain storage, but also the mature grain storage technology and equipment of above-ground granaries. Therefore, semi-underground granaries have become an important development direction for green and energy-efficient grain storage.

**Figure 11:**
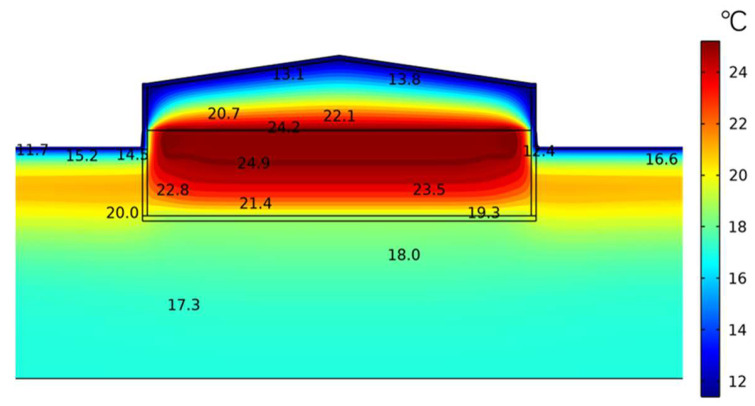
Simulated central vertical view of granary temperature field distributions for 31 December.

Figure 11 shows a central vertical view of the granary temperature field distributions for 31 December. The above-ground part of the grain pile showed the temperature distribution phenomenon of cold skin and hot core in the grain pile due to granary envelope heat transfer. The lowest temperature of the outer surface of the granary envelope near the ground surface was around 12.4 °C. The temperature of the grain pile near the inner surface of the granary envelope was around 21.4 °C, with large temperature gradients and large local temperature differences, which could increase the risk of condensation in the local zone near the granary wall. The temperature in the underground part of the grain pile remained high in cold winter due to the pile’s relatively low thermal conductivity. The highest temperature in the underground part of the grain pile was 24.9 °C, which exceeded the low-temperature grain storage threshold of 20 °C.

### 3.2. Effect of Micro Airflow Velocity Distribution on Heat Transfer in Granary

**Figure 12:**
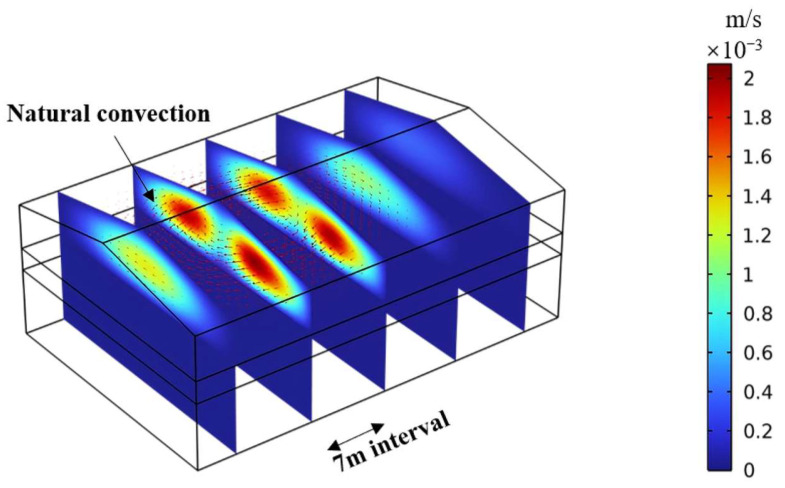
Longitudinal views of velocity field distributions of air layer in granary.

**Figure 13:**
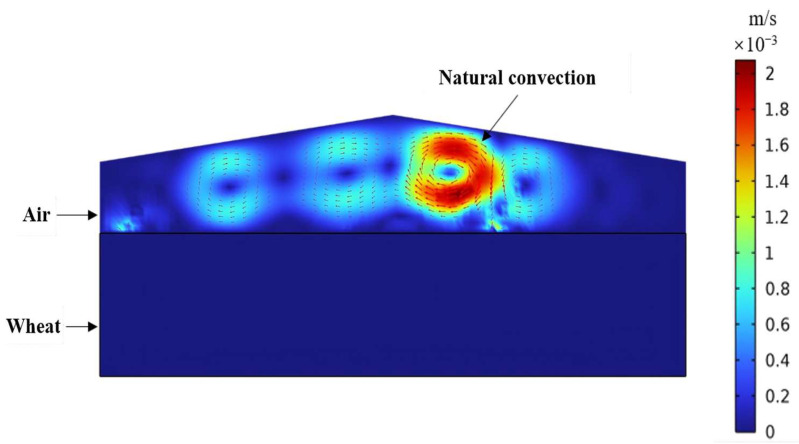
Vertical cross-section view of velocity field distributions of air layer in granary.

Natural convection in the air layer above the grain pile affects heat transfer and temperature field distributions in the underground granary. Figure 12 shows longitudinal views of velocity field distributions of the air layer in the granary. Figure 13 shows a vertical cross-section view of velocity field distributions of the air layer in the granary. The airflow in the air layer above the grain bulk was driven by thermal buoyancy. Because the vertical temperature gradient was small, the airflow velocities in the air layer above the grain pile were very low. In summer, heat transfer through the granary envelope caused the air temperature near it to rise, while heat transfer from the grain pile caused the air temperature near it to decrease. The buoyancy force caused by temperature differences creates a circulating airflow, which continuously transfers heat from the granary envelope to the grain pile, and vice versa. In winter, heat transfer through the granary envelope caused the air temperature near it to decrease, while heat transfer from the grain pile caused the air temperature near it to rise. Therefore, the micro-airflow velocity distribution in the granary can enhance heat exchange between the granary envelope and the grain pile.

### 3.3. Average Temperature of Grain Pile and Inner Surface of Granary Wall

**Figure 14:**
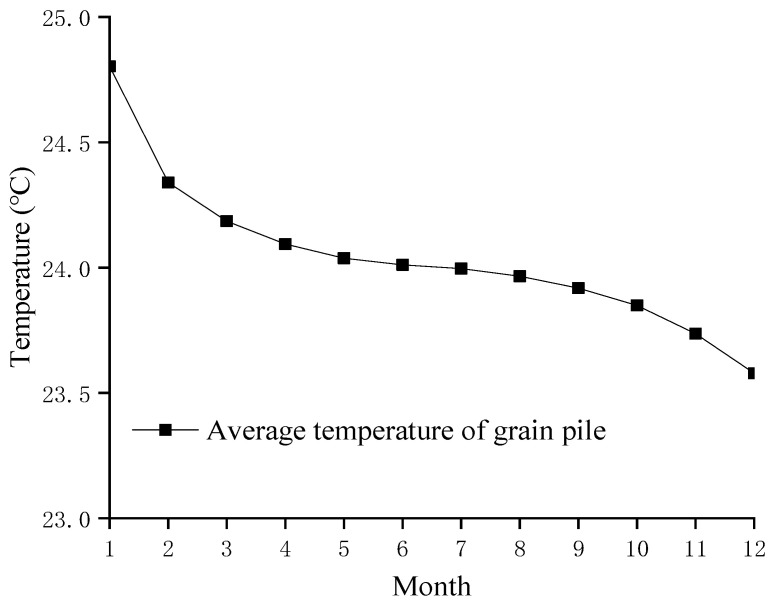
Predicted monthly average temperature of grain pile.

Figure 14 shows the predicted monthly average temperature of the grain pile. The changes in grain pile temperature in the semi-underground granary were determined by the temperature of the outside environment of the granary. The outside environment of the granary consisted of the outdoor air and surrounding soil environments. The coupled effects of outdoor air temperature and soil temperature had significant impacts on heat transfer through granary envelopes. The annual fluctuation range of the outdoor air temperature was relatively large, while the underground soil temperature remained relatively stable with smaller annual variations. Both outdoor air temperature and soil temperature had significant impacts on the temperature of the grain pile. In summer, the temperature of the outdoor air was higher than those of the soils and the grain pile. Heat was transferred from the outdoor air environment to the grain pile through the above-ground granary envelope. In the meantime, the surrounding low-temperature soil environment would absorb heat from the grain pile through the underground granary envelope. The heat losses caused by the surrounding soil and the heat gains from the outdoor air environment determine the temperature of the grain pile in the granary. The temperature of the grain pile in the granary showed a slower downward trend in summer, as shown in Figure 11. In winter, both the outdoor air temperature and surrounding soil temperature were lower than the grain pile temperature. Heat was transferred from the grain pile to the outdoor air environment and surrounding soil environment. The temperature of the grain pile in the granary showed a faster downward trend in winter, as shown in Figure 11. Owing to the thermal stability of the surrounding soil, the grain pile temperatures remained low with small annual fluctuations. The grain pile temperatures (23.5–24.8 °C) were lower than the low-temperature grain storage threshold of 20 °C.

**Figure 15:**
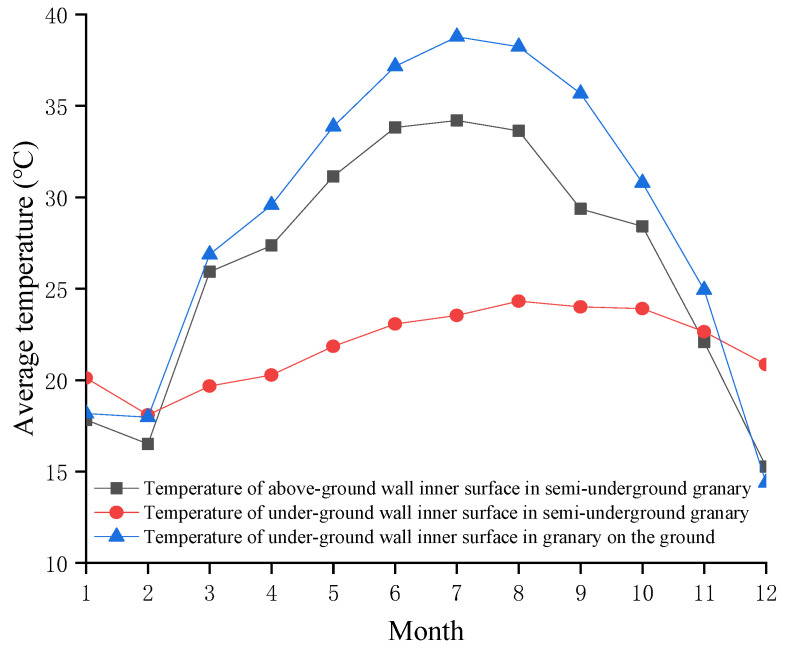
Predicted monthly average temperature of inner surface of granary wall.

Figure 15 shows the predicted monthly average temperature of the inner surface of the granary wall. Under the conditions of annual periodic variation in outdoor air temperature, the average temperature of the inner surface of the above-ground granary wall had a large temperature difference and a large temperature fluctuation range. The inner surface temperature of the above-ground granary wall was strongly influenced by the outdoor air temperature. The inner surface temperature of the above-ground granary wall ranged from 16.5 °C to 33.8 °C. As shown in Figure 15, the inner surface temperature of the above-ground granary wall was the lowest in winter and highest in summer. The inner surface temperature of the granary wall varies with the outdoor air temperature. The monthly average inner surface temperature of the above-ground granary wall varies with the monthly average outdoor air temperature. In contrast, the inner surface temperature of the underground granary ranged from 18.1 °C to 24.5 °C. The annual fluctuation of the inner-surface temperature of the underground granary wall was relatively small. The reason for this phenomenon was that thermal inertia of the surrounding soil was high, which attenuated the effect of external environment temperature fluctuations on the underground granary wall. As shown in Figure 15, the annual temperature variation of the above-ground granary wall exceeded 17 °C, while that of the underground granary wall was around 6.4 °C. This implies that the heat transfer through the semi-underground envelope results from the combined and non-linear coupled effects of the outdoor thermal environment and the stable soil geothermal environment.

## 4. Grain Temperature Control Method for Improving Grain Storage Safety of Granary

### 4.1. Floor Slab-Based Grain Temperature Control Method for Semi-Underground Granary

**Figure 16:**
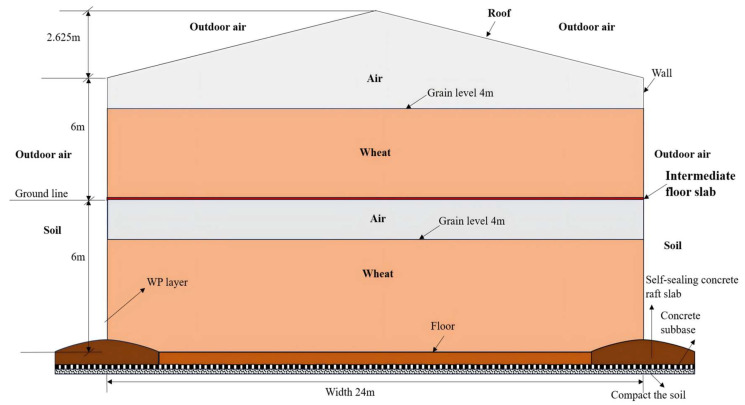
Schematic diagram of the floor slab-based grain temperature control method.

The shallow geothermal energy can maintain underground parts of the grain pile in the semi-underground granary at a lower grain storage temperature throughout the year, as shown in Figure 10 and Figure 11. The highest temperature in the underground part of the grain pile was 23.7 °Cand 24.9 °C in summer and winter, respectively. The highest temperature in the local zone of the grain pile near the ground surface was 32.3 °C and 19.6 °C in summer and winter, respectively. These values failed to meet the national grain storage temperature requirement for low-temperature grain storage (20 °C). To solve this problem of low-temperature grain storage, a floor slab-based grain temperature control method for semi-underground granaries is presented in this study to ensure that the highest temperature in the underground part of the grain pile is lower than 20 °C. In this new grain temperature control method, an intermediate floor slab is installed at the height of ground surface in the semi-underground granary. The intermediate floor slab separates the semi-underground granary into an above-ground section and an underground section, as shown in Figure 16. Numerical studies were conducted to investigate the actual grain temperature control effect of the new floor slab-based grain storage method. The height of the above-ground part of the grain pile in the granary was 4 m. The space above the upper granary section was an air layer. The height of he above-ground part of the grain pile in the above-ground section was 4 m. The zone between the floor slab and the grain pile in the underground section was a 2 m high air layer. The initial temperature of the above-ground grain pile was set at 25 °C, and that of the underground grain pile was set at 20 °C. The typical annual outdoor air temperature (Figure 5) and hourly solar radiation intensity (Figure 6) of Zhengzhou city in China were used to investigate the temperature field changes with the floor slab-based grain temperature control method for a semi-underground granary.

**Figure 17:**
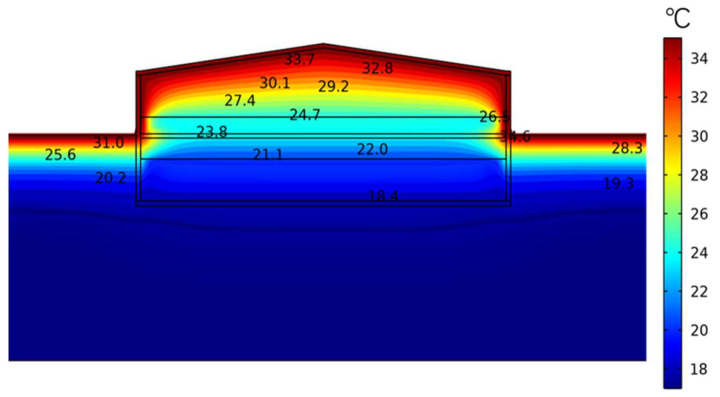
Simulated central vertical view of granary temperature field distributions for 17 August (with floor slab).

**Figure 18:**
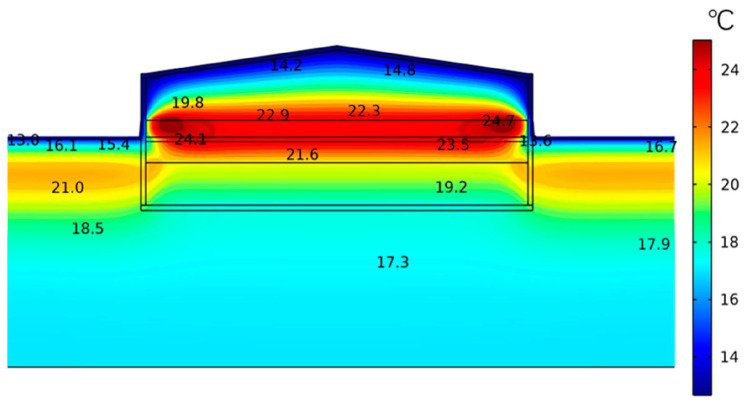
Simulated central vertical view of temperature field distributions for 31 December (with floor slab).

Figure 17 shows a central vertical view of the granary temperature field distributions for 17 August. The highest temperature in the local zone of the grain pile near the granary wall in the above-ground section was 27.4 °C. The maximum average temperature of the above-ground grain pile in the granary without an intermediate floor slab was 25.46 °C. The maximum average temperature of the grain pile in the above-ground section with an intermediate floor slab was 25.11 °C. Figure 18 shows a central vertical view of the granary temperature field distributions for 31 December. The temperature of the grain pile in the underground section remained below 20 °C throughout the whole year, meeting the requirements for low-temperature grain storage. The intermediate floor slab-based grain temperature control method can ensure that the underground section achieves natural quasi-low-temperature grain storage. The reason for this phenomenon is that the air layer in the underground section increases the vertical thermal resistance of the underground section of the granary. Less heat is transferred from the outdoor air environment into the grain pile in the underground section of the granary. However, the thermal conductivity of the intermediate floor slab is higher than that of the grain pile. An obvious thermal bridge effect occurs at the position of the intermediate floor slab, which localizes heat concentration and rapid heat transfer. To mitigate the adverse effects of thermal bridge heat transfer, a thermal insulation layer should be added to the granary envelope near the intermediate floor slab. In the design optimization and engineering applications for semi-underground granaries, an intermediate floor slab should be included to improve grain storage security levels.

### 4.2. Embedded-Pipe Wall-Based Grain Temperature Control Method for Granary

**Figure 19:**
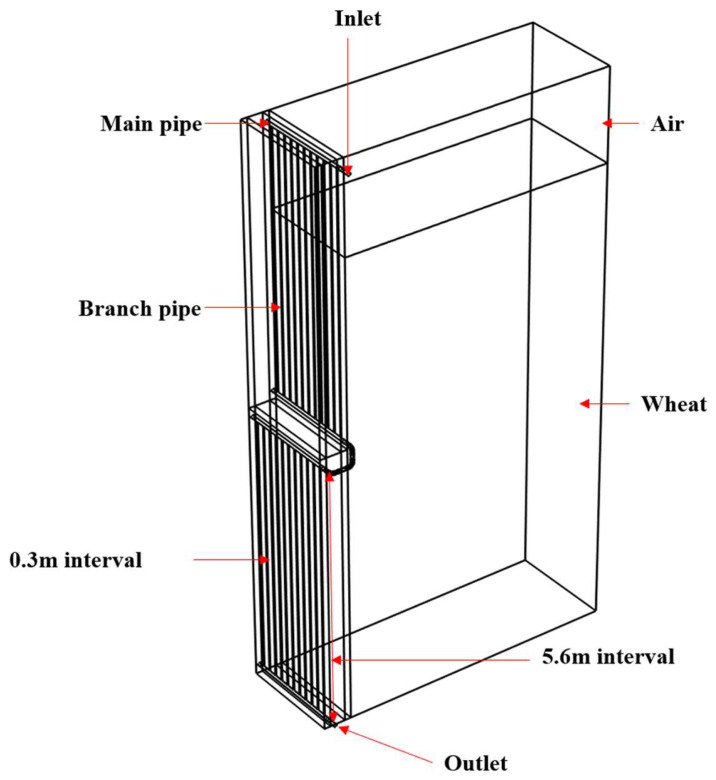
Schematic diagram of water pipe arrangement in the granary wall.

The above-ground part of the granary showed the temperature distribution phenomenon of hot skin and cold core in the grain pile due to granary envelope heat transfer. The highest temperature in the local zone of the grain pile near the ground surface was 32.3 °C. The temperature of the grain pile in the zone near the ground surface had large temperature gradients and large local temperature differences. To address the local temperature rise issue in the grain pile near the granary wall, an embedded-pipe wall-based grain temperature control method for semi-underground granaries is presented in this study to reduce the local temperature of the grain pile near the granary wall and improve the safety level of grain storage. The water pipes were arranged in the granary wall in a main-branch-main pipe configuration, as shown in Figure 19. The vertical pipe spacing was 0.3 m, and the horizontal pipe spacing was 5.6 m. The vertical main water pipe diameter was 40 mm, and the horizontal branch water pipe diameter was 12 mm. The material of the pipe was plastic. The inlet water temperature was set to 18 °C. The inlet water flow rate was set to 0.5 m/s. The initial grain pile temperature was set to 28 °C. Numerical studies were conducted to investigate the actual temperature control effect of the new embedded-pipe wall-based grain temperature control method.

**Figure 20:**
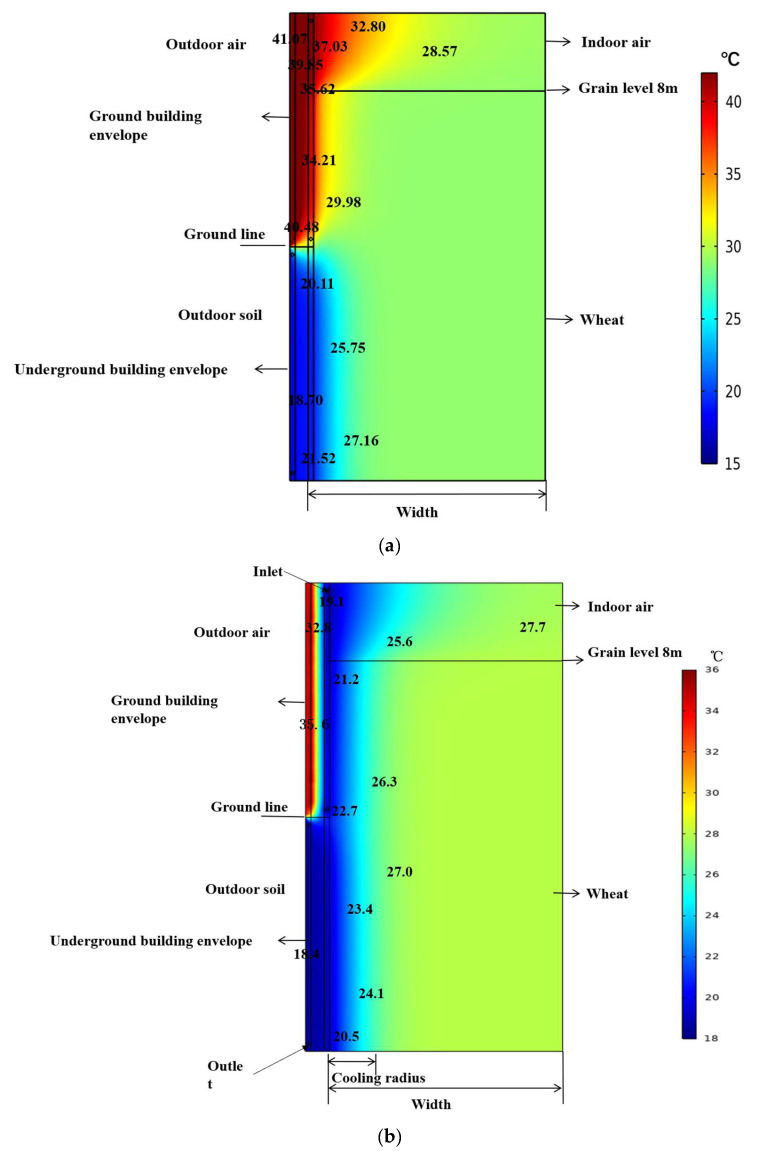
Central vertical view of temperature field distributions in granary. (**a**) Without embedded water pipe; (**b**) With embedded water pipe.

Figure 20 shows a central vertical view of temperature field distributions in the grain pile near the granary wall with and without embedded water pipes. After 7 days of continuous operation of the embedded-pipe cooling system in the granary wall, the local zone temperature of the grain pile near the granary wall dropped significantly. The results of simulations showed that the above-ground section of the grain pile near the granary wall was strongly affected by outdoor air temperature changes. The temperature difference between the above-ground section and underground section of the grain pile in the granary was large. The large temperature difference tends to cause condensation in the grain pile, which is not conducive to safe grain storage in semi-underground granaries. In contrast, the granary wall with embedded water pipes decreased the grain temperature in the local zone of the grain pile near the granary wall significantly. The temperature difference of the grain pile near the above-ground granary wall and underground granary wall was almost eliminated by using the embedded-pipe cooling system.

**Figure 21:**
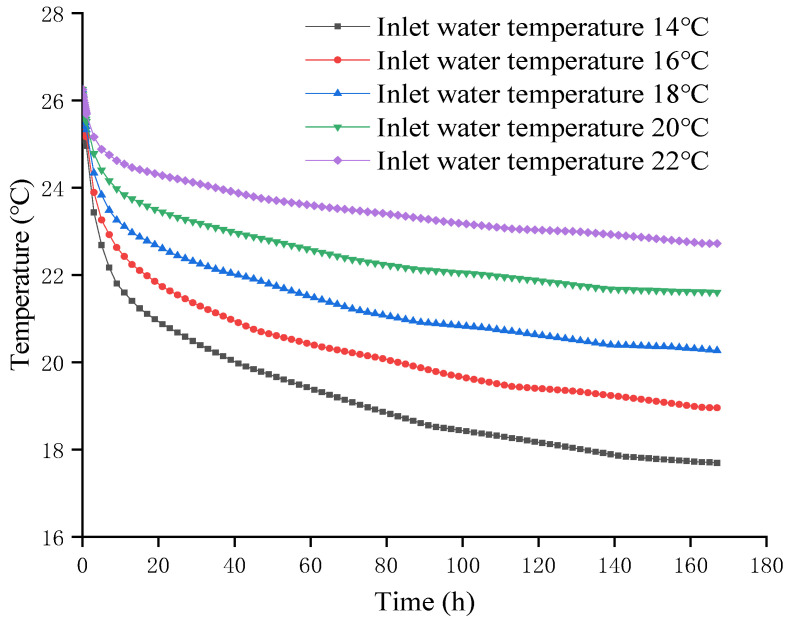
Inner surface temperature of wall with different inlet water temperatures.

The inlet water temperature of the embedded pipes affected the temperature distribution of the granary wall and grain pile. Figure 21 shows the average inner surface temperature of the granary wall with different inlet water temperatures. The average inner surface temperature of the granary wall for inlet water temperatures of 14, 16, 18, 20, and 22 °C was 17.7, 18.9, 20.2, 21.7, and 22.8 °C, respectively. The lower the inlet water temperature, the lower average inner surface temperature of the granary wall. The rate of the drop in the average inner surface temperature of the granary wall was first rapid and then slow. Thus, the inlet water temperature plays a crucial role in controlling the grain pile temperature. Because the thermal conductivity of the grain pile is low, the cooling effect of the embedded-pipe cooling system is confined to a limited range. As shown in Figure 20b, beyond the cooling effect, the grain pile is not influenced by the embedded-pipe cooling system.

### 4.3. Floor Burial Depth-Based Grain Temperature Control Method for Granary

**Figure 22:**
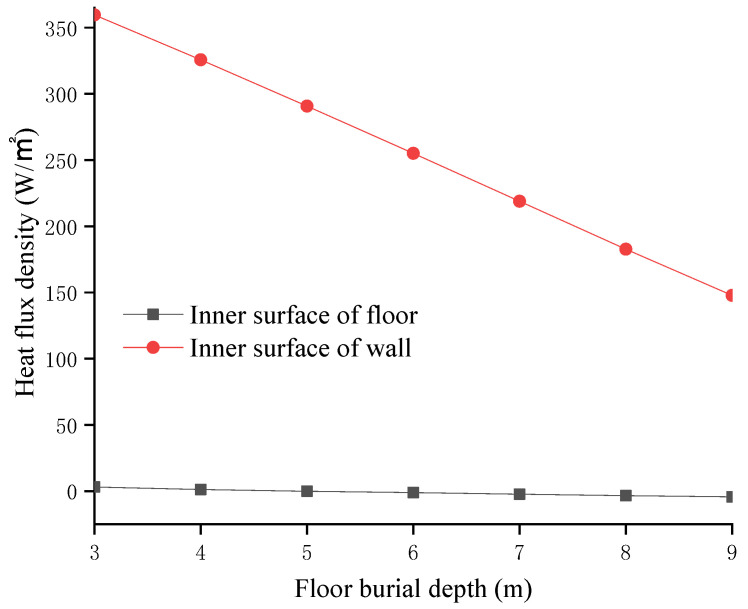
Heat flux density of inner surface of envelope for different floor burial depths.

The temperatures of grain piles in semi-underground granaries are influenced by the coupled effects of outdoor air temperature and soil temperature. The influence of the floor burial depth of a semi-underground granary on the grain pile temperature field was studied using the steady-state heat transfer simulations. Affected by heat exchange between the ground surface and the outdoor air environment, the temperatures at different soil depths differed significantly from one another. As the soil depth increased, the influence of the outdoor air environment on the soil temperature gradually decreased until it became negligible [41]. In this study, semi-underground granary floor burial depths of 3, 4, 5, 6, 7, 8, and 9 m were considered. The outdoor air temperature was set at 38.8 °C according to the national design standard of China. The thermal conductivity, density, and specific heat capacity of concrete were set to 1.74 W/(m·K), 2500 kg/m^3^, and 920 J/(kg·K), respectively [42]. The constant-temperature layer was located at a depth of 17 m. The temperature of the constant-temperature layer was 17 °C. The soil thermal conductivity was set to 0.941 W/(m·K). The soil density was set to 880 kg/m^3^. The soil specific heat capacity was set to 1170 J/(kg·K). The indoor temperature of the granary was set to 20 °C. The convective heat transfer coefficient on the outer surface of the envelope was set to 23 W/(m^2^·K). The outdoor wind speed was set to 3 m/s. The heat transfer performance of the envelope was quantified by the average heat flux density of the inner surface of granary walls. Figure 22 shows the heat flux density of the inner surface of the envelope for different floor burial depths of semi-underground granaries. The average heat flux densities of the inner surface of the granary floor for 3, 4, 5, 6, 7, 8, and 9 m floor burial depths were 3.11, 1.29, −0.02, −1.00, −2.26, −3.32, and −4.28 W/m^2^, respectively. As the floor burial depth increased from 3 to 9 m, the average heat flux density of the inner surface of the granary floor decreased from 3.11 W/m^2^ to −4.28 W/m^2^. The average heat flux densities of the inner surface of the granary wall for 3, 4, 5, 6, 7, 8, and 9 m floor burial depths were 359.94, 325.68, 290.73, 255.12, 218.84, 182.72, and 147.87 W/m^2^, respectively. As the floor burial depth increased, the average heat flux density of inner surface of the granary wall decreased from 325.68 W/m^2^ to 182.72 W/m^2^. The change in inner surface heat flux density of the granary wall was far greater than that of the granary floor. This phenomenon indicates that the floor burial depth of a semi-underground granary is a key influencing factor of granary wall heat transfer. A larger floor burial depth can reduce the average grain pile temperature and improve the energy-saving performance of a semi-underground granary.

### 4.4. Envelope Insulation-Based Grain Temperature Control Method for Granary

**Figure 23:**
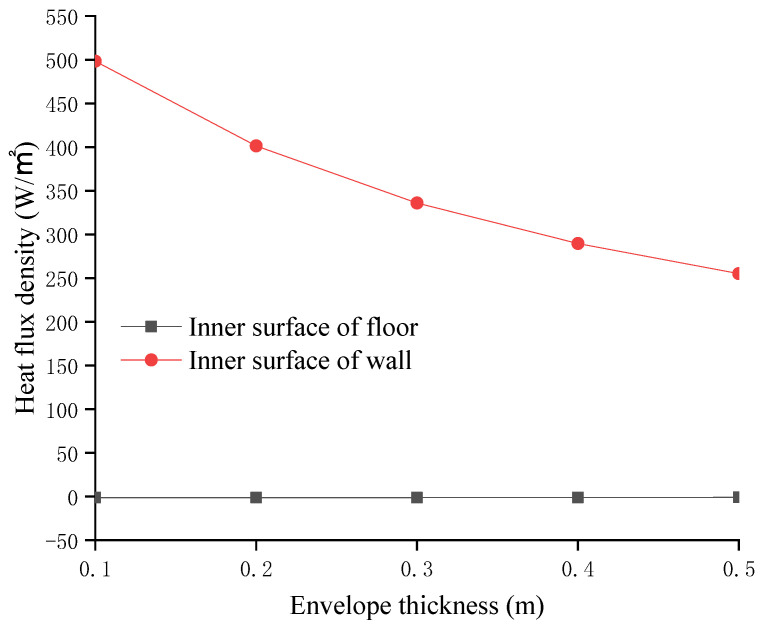
Average heat flux density of inner surface for different envelope thicknesses.

The envelope insulation has important effects on the envelope heat transfer of a semi-underground granary. The granary envelope insulation varies with different building material types and envelope thicknesses. There are various types of building materials, making it impossible to comprehensively analyze the impacts of building material types on the thermal resistance of building envelopes. In this study, the influence of envelope insulation on the grain storage temperature in the semi-underground granary was studied by using the steady-state heat transfer simulations and through changing granary envelope thicknesses. Simulation scenarios of reinforced concrete floors and walls with thicknesses of 0.10, 0.20, 0.30, 0.40, and 0.50 m were considered to investigate the effects of envelope insulation on the grain storage temperature in the semi-underground granary. Figure 23 shows the average heat flux density of the inner surface of the granary envelope for different floor and wall thicknesses. The average heat flux densities of the inner surface were −1.35, −1.33, −1.25, −1.17, and −1.00 W/m^2^ for granary floors with a thickness of 0.10, 0.20, 0.30, 0.40, and 0.50 m, respectively. The average heat flux densities of the inner surface were 498.20, 401.29, 335.88, 289.59, and 255.12 W/m^2^ for granary walls with a thickness of 0.10, 0.20, 0.30, 0.40, and 0.50 m, respectively. The average heat flux densities of the inner surface of the granary envelope decreased with increasing envelope thickness. Granary wall thickness had a significant impact on the thermal performance of the wall and grain pile temperature field. However, granary floor thickness had a small effect on the thermal performance of the wall and grain pile temperature field. In actual semi-underground granary buildings, the wall thickness should be determined basing on structural safety, thermal insulation requirements, and economic considerations.

The purpose of this paper is to propose energy-saving grain pile temperature control methods that can reduce the energy consumed for grain temperature control through mechanical ventilation and air conditioning in the granaries. In actual granaries, the temperature of the above-ground grain pile is allowed to exceed 20 °C. However, when the grain storage temperature exceeds the upper threshold of grain temperature control, a mechanical ventilation system will be used to control the grain pile temperature. The upper limit of grain temperature control is determined based on the specific requirements for grain temperature control in actual granaries. The energy consumed by the presented grain pile temperature control methods was lower than that of the above-ground granary, which uses mechanical ventilation systems to control grain storage temperature. To quantitatively assess the energy-saving potential of the proposed grain temperature control methods, the annual cumulative reduction in heat load was estimated based on the simulated inner-surface heat flux density. Taking the case of the envelope insulation-based grain temperature control method for granaries in Section 4.4 as an example, increasing the wall thickness from 0.1 m to 0.5 m reduces the inner-surface heat flux density from 498.20 W/m^2^ to 255.12 W/m^2^ (Figure 23), resulting in a net decrease of about 243.08 W/m^2^. The total wall area of the granary concerned was about 1440 m^2^. Accordingly, the energy-saving performance of the envelope insulation-based grain temperature control method can be determined based on the simulation results for the reduction in heat flux density of the envelope inner surface in the granary. The reduction of annual granary energy consumption is about 2134.69 kWh. A significant reduction in heat load will decrease the electricity cost for mechanical ventilation systems in granaries. Comparisons with other works in the literature show that the current findings for grain temperature fields and the energy-saving performance of grain storage temperature control methods in semi-underground granaries are supported by many existing studies. Experiments and numerical simulations conducted by Jin et al. [22,24,25] found that a semi-underground granary could significantly reduce the grain storage temperature and energy consumption compared with above-ground granaries.

## 5. Conclusions

Semi-underground granaries represent a new type of energy-saving grain storage facility that can use shallow geothermal energy to reduce energy consumption during grain storage. The presented grain temperature control methods only require a small increase in initial investment for the construction of semi-underground granaries, but they can significantly reduce the long-term energy consumption of mechanical ventilation systems by utilizing shallow geothermal energy. Temperature fields of the grain pile in the semi-underground granary have been numerically studied by using an experimentally verified COMSOL model. Moreover, multiple grain storage temperature control methods have been presented to improve grain storage safety, including intermediate floor slab, embedded-pipe wall, floor burial depth, and envelope insulation. The main conclusions are as follows:

(1) The presented collaborative simulation method combining steady-state heat transfer and dynamic heat transfer can provide a useful tool for numerical studies on temperature field and grain temperature control methods for semi-underground granaries.
(2) There was significant spatial heterogeneity in the temperature field distributions of the grain pile in the semi-underground granary, as shown in Figure 10 and Figure 11.
(3) The floor slab-based grain temperature control method can achieve natural low-temperature grain storage in the underground section, effectively reducing the local maximum temperature from 23.7 °C to below 20 °C.
(4) The embedded-pipe wall-based grain temperature control method can solve the problem of local temperature rise near granary walls, decreasing the peak temperature of grain piles near the granary wall from 32.6 °C to 27.7 °C.
(5) Increasing granary wall thickness and wall insulation can decrease the annual temperature fluctuation amplitude of the grain pile in the semi-underground granary. Increasing granary wall thickness from 0.1 m to 0.5 m reduces the inner-wall heat flux by about 243.1 W/m^2^.

Although this study addressed the problems of temperature fields and grain storage temperature control methods for semi-underground granaries, only one representative granary building type and outdoor atmospheric environment were considered. The influences of building type, moisture migration, and grain moisture content on grain storage temperature control should be addressed in future studies.

## Figures and Tables

**Table 1:** Thermophysical parameters of main building materials.

Materials	Density (kg/m^3^)	Thermal Conductivity (W/(m·K))	Heat Capacity (J/(kg·K))	Porosity
Wheat [27]	750	0.15	1780	0.43
Air [27]	1.225	0.024	1006.43	/
Reinforced concrete [28]	2500	1.74	920	/
Soil [29]	880	0.941	1170	0.385
Cement mortar [28]	1800	0.93	1050	/
Concrete [27]	2300	0.79	1000	/

**Table 2:** Relative error of different grid schemes.

Mesh Schemes	Grid Quantity	Relative Error
Mesh 1	1,055,490	0.94%
Mesh 2	2,062,671	0.51%
Mesh 3	2,479,938	0.14%
Mesh 4	3,077,995	Base

## Data Availability

The original contributions presented in this study are included in the article. Further inquiries can be directed to the corresponding author.
